# Exosomal Sonic Hedgehog derived from cancer‐associated fibroblasts promotes proliferation and migration of esophageal squamous cell carcinoma

**DOI:** 10.1002/cam4.2873

**Published:** 2020-02-07

**Authors:** Guiping Zhao, Hengcun Li, Qingdong Guo, Anni Zhou, Xingyu Wang, Peng Li, Shutian Zhang

**Affiliations:** ^1^ Department of Gastroenterology National Clinical Research Center for Digestive Disease Beijing Digestive Disease Center Beijing Key Laboratory for Precancerous Lesion of Digestive Disease Beijing Friendship Hospital Capital Medical University Beijing P. R. China

**Keywords:** cancer‐associated fibroblasts, esophageal squamous cell carcinoma, exosomes, Sonic Hedgehog

## Abstract

Esophageal squamous cell carcinoma (ESCC) is one of the most common and aggressive malignancies in China. Cancer‐associated fibroblasts (CAFs) can actively communicate with and stimulate tumor cells, thereby contributing to the development and progression of tumors. Yet, whether CAFs‐derived exosomes have a role in the progression of ESCC is largely unknown. Here, we find that Sonic Hedgehog (SHH) is highly expressed in CAFs lysis solution, conditioned medium of cultured CAFs (CAF‐CM) and CAFs‐derived exosomes, and esophageal cancer cell lines educated by CAF‐CM and CAFs‐derived exosomes can improve their growth and migration abilities in vitro and in vivo. Besides, those effects can be partly neutralized by cyclopamine, inhibitor of the Hedgehog signaling pathway. Thus, our research elucidates the crucial role of CAFs‐derived exosomes in the growth and progression of ESCC, and may open up new avenues in the treatment of ESCC.

## Graphical abstract

Exosomal Sonic Hedgehog derived from cancer‐associated fibroblasts promotes proliferation and migration of esophageal squamous cell carcinoma and this effect can be partly neutralized by the inhibitor of the Hedgehog signaling pathway.

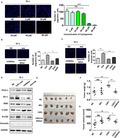

## 1 INTRODUCTION

Esophageal squamous cell carcinoma (ESCC) is one of the most common and aggressive malignancies in China.^1^ Despite recent advances in the diagnosis and treatment of ESCC, the final prognosis remains unsatisfactory. The overall 5‐year survival rate of advance ESCC is about 10%‐20%.^2^, ^3^ The absence of early symptoms, high rates of local invasion, and lymphatic metastasis largely contributes to the unfavorable prognosis of ESCC, thus revealing the underlying mechanism of ESCC and discovery of new drug targets is of vital importance.

Accumulating evidence^4^, ^5^ suggests that the malignant biological behaviors of cancers depend not only on the cancer cells themselves, but closely on the regulation of the tumor microenvironment. Cancer‐associated fibroblasts (CAFs), the most common stromal cell type of the microenvironments, can actively transfer information with and stimulate tumor cells, thereby promoting the development and progression of tumors.^6^ Previous studies have successfully isolated CAFs and normal fibroblasts (NFs) from tumor tissues or normal tissues, and the expression of myofibroblast marker α‐SMA in CAFs is significantly higher than that in NFs.^7^ A well‐recognized theory indicates that CAFs can secrete a large number of soluble factors, including growth factors, inflammatory cytokines, and chemokines, and promote the progression and metastasis of cancer. Yu suggests that CAFs in breast cancer can induce the epithelial–mesenchymal transition (EMT) of cancer cells through the activation of paracrine TGF‐β signaling.^8^ Liu et al indicates that CAF‐secreted chemokines promote hepatocellular carcinoma (HCC) metastasis through the activation of TGF‐β pathways.^9^ There is a well‐accepted hypothesis that CAFs can affect the proliferation, migration, invasion, and metastasis in ESCC. However, the underlying molecular mechanisms have not been fully clarified.

Exosomes, firstly introduced by Trams et al in 1981, are small (30‐200 nm), lipid bilayer membrane vesicles which contain and transfer variety of bioactive lipids, proteins, mRNAs, and noncoding RNAs.^10^, ^11^ Those extracellular vesicles can be identified from various body fluids including amniotic fluid, ascites, saliva, serum, plasma, urine, as well as the culture medium of cells.^12^, ^13^, ^14^ The roles of exosomes in the development of tumors have gained extensive attention in recent years, and increasing evidence reveals that exosomes are involved in many tumor‐related pathways, such as apoptosis, invasion, migration, and metastasis.^15^, ^16^, ^17^ Li et al found that CAFs‐derived exosomes can promote EMT in ovarian cancer cells via the activation of TGFβ1 signaling pathway.^15^ Zhang et al indicates that exosomal EGFR derived from gastric cancer cells could facilitate the formation of a liver‐like microenvironment and promote the specific metastasis to liver.^16^

The Hedgehog signaling pathway, a mediator of proliferation and differentiation in vertebrate embryogenesis, has been reported to be implicated in the development of several cancers including medulloblastoma, pancreatic cancer, gastric cancer, and breast cancer.^18^, ^19^, ^20^ There exist three types of Hedgehog homologs in the mammalian signaling pathway family, among which Sonic Hedgehog (SHH) is best investigated. Like TGFβ1, SHH proteins are also secreted signaling molecules which have several effects on recipient cells. SHH can activate the Hedgehog signaling of the recipient cell by binding to the Patched (Ptch), a 12‐transmembrane protein on the cell membrane. Some studies show that the activation of SHH signaling can facilitate the proliferation, invasion, or angiogenesis of the tumors,^21^, ^22^ however, most studies stress their emphasis on the effects of SHH as a proto oncoprotein of the tumor cells,^23^, ^24^, ^25^ and data on the importance of SHH in the CAFs—tumor cell crosstalk is sparse at best.

In recent years, the role of SHH signaling in esophageal cancer has gained extensive attention. As far as we know, the activation of SHH signaling affects all aspects of tumorigenesis, development, and metastasis of esophageal cancers. It is reported that the elevated expression of SHH and its target genes is not uncommon in most kinds of esophageal cancers.^25^ Yang et al found that the activation of SHH signaling is an early molecular event in esophageal cancers,^26^ which indicates that it may play a role in the tumorigenesis of esophageal cancer. Besides, several studies illustrated that the activation of Hh signaling is essential for the progression and metastasis of esophageal cancer. Wang et al proved that the overexpression of Gli, target gene of SHH, promoted EMT in human esophageal adenocarcinoma.^27^ In ESCC, the expression of Gli1 is reported to be closely associated with the tumor progression and lymph node metastasis.^28^ Besides, some studies indicated that the target gene of Hh, including Gli1 and PTCH1, can be identified as an independent adverse prognostic factor in ESCC.^26^, ^29^ Collectively, the activation of SHH signaling is closely linked to the oncogenesis and development of esophageal cancers, researches about their correlation may offer new ideas for the treatment of ESCC.

Hitherto, the stromal cell‐derived molecular determinants that modulate ESCC progression have not been fully characterized. In this study, we find that SHH is highly expressed in CAFs lysis solution, conditioned medium of cultured CAFs (CAF‐CM) and CAFs‐derived exosomes. More importantly, CAF‐CM and CAFs‐derived exosomes can substantially improve the growth and migration abilities of ESCC cell lines in vitro and in vivo. Additionally, those effects can be partly neutralized by Cyclopamine, an inhibitor of the Hedgehog signaling pathway. Thus, our research elucidates the crucial role of CAFs‐derived exosomes in the growth and progression of ESCC, and may open up new avenues for the treatment of ESCC.

## 2 MATERIALS AND METHODS

### 2.1 Isolation and culture of CAFs and NFs

To obtain the stromal fibroblasts of ESCC, we isolated primary cancer tissues and normal tissues at least 5 cm from the tumor margin from six ESCC patients at Beijing Friendship Hospital (Beijing, China) between August 2017 and April 2018. Six patients had undergone esophagectomy but not received chemotherapy before surgical operation. This study was approved by the ethics committee of Beijing Friendship Hospital, and all subjects had signed written consents before participating in this study. All experiments were conducted according to the approved guidelines and regulations.

The ESCC tissues used for isolate stromal fibroblasts were diagnosed as invasive ESCC according to the American Joint Committee on Cancer (AJCC) staging manual (seventh edition).^30^ All sample tissues used for isolation were immediately preserved and transferred on ice, and the isolation of fibroblasts was performed within 2 hours after collection.

Briefly, the tissue samples were chopping and digested with 500 mg/mL Collagenase II (Beijing Solarbio Science & Technology) at 37℃ for 2.5 hours. Then the cells were gathered and cultured in RPMI‐1640 medium added with 15% fetal bovine serum (FBS; Invitrogen) and 1% Penicillin‐Streptomycin‐Gentamicin Solution for 3 hours. Remove all the supernatant (mostly epithelial cells and dead cells) and the remaining fibroblasts were seeded in RPMI‐1640 medium containing 15% FBS and 1% Penicillin‐Streptomycin‐Gentamicin Solution. After cultured for two to three passages, a homogeneity of stromal fibroblasts can be formed and the CAFs or NFs were cultured in RPMI‐1640 medium containing 10% FBS. All the stromal fibroblasts used in our experiments were at less than eight passages.

### 2.2 Culture of cell lines

Esophageal squamous cell carcinoma cell lines EC109 and TE‐1 were generously provided by Cancer Institute and Hospital, Chinese Academy of Medical Sciences. EC109 and TE‐1 cells were both cultured in RPMI‐1640 medium added with 10% FBS, with the temperature of 37°C and 5% CO_2_.

### 2.3 Exosomes isolation and labeling

Cancer‐associated fibroblasts and NFs were cultured in RPMI‐1640 medium containing 10% exosome‐depleted FBS before exosomes isolation for 48 hours. Exosomes were isolated from CM of CAFs and NFs by differential ultracentrifugation, according to the previous publications. In brief, we first removed the cells and other debris by centrifugation at 300 and 3000 *g* for 30 minutes, respectively. Then, we removed the shedding vesicles and other bigger‐sized vesicles by centrifugation at 10 000 *g* for 30 minutes. After removing the precipitations, the supernatant was centrifuged at 120 000 *g* for 70 minutes twice. We then resuspended the exosome pellets with 5‐mL phosphate‐buffered saline (PBS) and centrifuged again at 120 000 *g* for 70 min to remove the remaining proteins. Finally, the exosomes were resuspended and preserved in PBS at −80°C until further analyses. Then we measured the concentration of the exosomes using BCA method according to the manufacturer's instructions (Thermo Scientific). Exosomes isolated from CM of CAFs were labeled using PKH67 Green Fluorescent Cell Linker Mini Kit as recommended by the manufacturer (Sigma Aldrich).

### 2.4 Transmission electron microscopy

The morphology of exosomes was detected by transmission electron microscopy (TEM). First, we diluted and mixed the exosomes with PBS, and then the diluted exosomes were put on copper grids for 1 minute. After staining the grids with 1% (v/v) uranyl acetate in ddH_2_O, the samples were detected and analyzed by TEM (Hitachi).

### 2.5 NanoSight particle tracking analysis

Exosomes derived from CAFs or NFs were diluted and mixed well with PBS. Exosomes were slowly injected into the sample chamber of NanoSight LM10 instrument to avoid small air bubbles. And we detected and analyzed the concentration and size distribution of the exosomes by NTA instrument and NTA analytical software.

### 2.6 Western blot analysis

The expression of the proteins was measured by western blotting analysis and the GAPDH was used as control. Protein extraction from cells or exosomes was performed using radio immunoprecipitation assay buffer. The concentration of the proteins was measured using BCA method according to the manufacturer's suggestions (Thermo scientific Pierce). Equal amount of proteins (25 μg) was loaded to assess the expression of specific protein. The proteins were separated by a 10% SDS‐PAGE gel and transferred to a PVDF membrane (Millipore) that was socked in methanol for 2 minutes before using. The membrane was then blocked in 5% nonfat milk and rinsed before incubated with primary antibodies overnight at 4°C. Antibodies against CD‐63, CD‐9, GM130, GLI1, and TSG101 were purchased from ABCAM (Abcam), and antibodies against E‐cadherin, vimentin, and N‐cadherin from Cell Signaling Technology. Antibodies against SHH, PTCH1, and SMO were purchased from Proteintech. After washing, the blots were incubated with the secondary antibodies at 37°C for 2 hours and rinsed for three times before visualized by an ECL plus system (Beyotime).

### 2.7 Enzyme‐linked immunosorbent assay

The expressions of TGF‐β1 and SHH in exosomes and in CMs of CAFs and NFs were measured by enzyme‐linked immunosorbent assay (ELISA). The TGF‐β1 and SHH ELISA kits (eBioscience) were used according to the manufacturer's instructions.

### 2.8 Cell proliferation assay

A density of 2000 TE‐1 or EC109 cells were seeded in each well of a 96‐well plate and treated with or without exosomes. Viability of the cells was measured at the time point of 0, 24, 48, and 72 hours using MTS reagent, CellTiter 96^®^ Aqueous One Solution Cell Proliferation assay (Promega). The optical density at 490 nm was detected using enzyme‐labeled meter (Spectramax M3; Molecular Devices) after incubated at 37°C for 2 hours. Three independent tests were conducted for the cell proliferation assay.

### 2.9 Wound‐healing assay

In wound‐healing assay, TE‐1 or EC109 cells were seeded in 6‐well plates and grown until 100% confluent before experiments. The wound was created by a 20‐µL pipette tip in the confluent monolayer at the center of culture plates. The wells were washed with PBS buffer to remove the nonadherent cells scratched by the pipette tip. Then the cells were cultured with culture medium containing exosomes or not. The images of the wound were captured at 0 and 24 hours after operation. The migratory distance was detected using ImageJ software.

### 2.10 Cell migration and invasion assay

Cell migration and invasion assay of TE‐1 and Ec109 cells were performed using Matrigel‐coated Transwell and Transwell inserts (Becton Dickinson). Briefly, 1 × 10^5^ cells mixed well in 500 µL serum‐free medium were inoculated in the upper chamber of the 24‐well plates, and 750 µL medium containing 10% FBS with or without exosomes was added into the lower chamber. Twenty‐four hours later, cells on the upper surface of the membrane were removed and the migrated cells or the invading cells penetrating the membrane were fixed with methanol and stained with mounting medium containing DAPI (Vector Laboratories, Inc). The slides were then scanned and photographed by fluorescence microscope (Olympus). The number of cells penetrating the membrane were analyzed by the ImageJ software.

### 2.11 Immunofluorescence assays

TE‐1 and Ec1009 cells were grown on slides to 50% confluence before fixed with 4% PFA for 15 minutes at room temperature. After rinsed with PBS buffer, the slides were incubated in 0.3% Triton X‐100 for 15 minutes to disrupt the cell membrane. The cells were blocked with 5% bovine serum albumin for 45 minutes, and then incubated with the primary antibodies (α‐SMA or Fibronectin) at 4°C overnight. Rinsing the slides with PBS buffer to remove the remaining primary antibodies and the cells were incubated with the Fluorescent secondary antibody mixture (Alexa Fluor 488 goat anti‐mouse IgG, Life Technologies; Alexa Fluor 568 goat anti‐mouse IgG, Life Technologies) at room temperature for 2 hours. The slides were then rinsed with PBS for three times and with DDW for one time before stained with mounting medium containing DAPI. The immunofluorescence images were obtained using confocal microscopy (IX83, FLUOVIEW FV1200; Olympus).

### 2.12 Animal studies

Twenty‐five male BALB/c nude mice aged 5‐6 weeks were randomized into four groups (six mice/group). The mice were inoculated subcutaneously with untreated EC109 cells (5 × 10^6^ cells in 100 μL of PBS) alone or coinjected with 1 × 10^6^ NFs/CAFs. One week later, PBS or 50 mg/kg Cyclopamine (Selleckchem) was injected into the tumor every 2 days for eight times. After 1 month, all the mice were executed and the tumors were surgically removed, photographed, measured, and weighed.

### 2.13 Statistical analysis

GraphPad Prism 5.0 software was performed for the statistical analysis. Data were presented as mean ± SE of at least three independent experiments. Independent‐sample *t* test was used to analyze the differences between two different groups and statistical differences between three groups were analyzed by one‐way ANOVA. A value of *P* < .05 was considered statistically significant unless otherwise clarified.

## 3 RESULTS

### 3.1 Isolation and identification of CAFs and NFs

**Figure 1:**
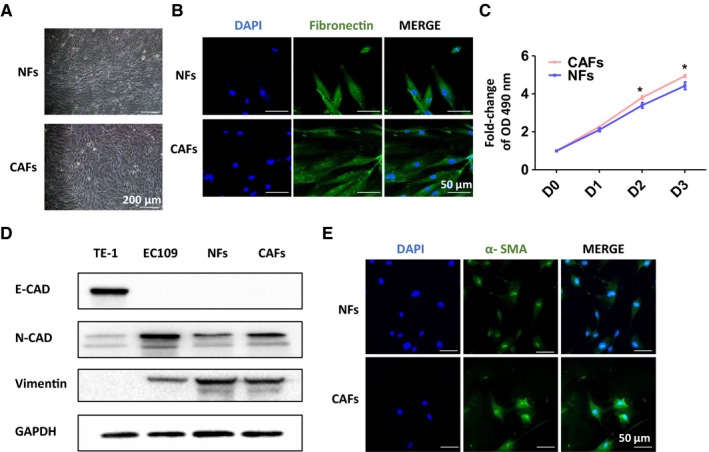
Isolation and identification of cancer‐associated fibroblasts (CAFs) and normal fibroblasts (NFs). A and B, The cell morphology of CAFs and NFs was observed; grew as a fibroblast‐like, spindle‐shaped morphology of cells were represented under light microscope or Confocal microscopy. C, Cell growth assay of CAFs and NFs. D, Western blot analyses of the expression of mesenchymal marker, N‐cadherin, vimentin and epithelial and endothelial marker, E‐cadherin in those stromal cells. E, IF assay of the expression of myofibroblast marker α‐SMA in NFs and CAFs

To investigate the role of CAFs in the microenvironment of ESCC, we firstly isolated and cultured primary NFs and CAFs from ESCC tissues. In our study, CAFs and NFs were successfully isolated and cultured from six pairs of ESCC tumor tissues and adjacent normal tissues. Three to four passaging after isolation, uniform fibroblasts started to grow and the morphological features of the NFs and CAFs were observed under light microscopy and Confocal microscopy. As shown in Figure 1A,1, both stromal cells grew as a fibroblast‐like, spindle‐shaped morphology and no obvious morphological difference was observed between them. The MTS assay showed that the CAFs proliferated more efficiently than NFs (Figure 1C). Both NFs and CAFs expressed the mesenchymal marker, N‐cadherin and vimentin, but not the common markers of epithelial and endothelial cells, E‐cadherin (Figure 1D). Consistent with previous reports, CAFs isolated from tumor tissues expressed higher levels of myofibroblast marker a‐SMA than NFs (Figure 1E). These data suggested that the fibroblasts we obtained from ESCC tissues exhibited the characters of CAFs and are more activated myofibroblasts than NFs.a

### 3.2 The CM of CAFs enhanced the migration ability of ESCC cell lines

**Figure 2:**
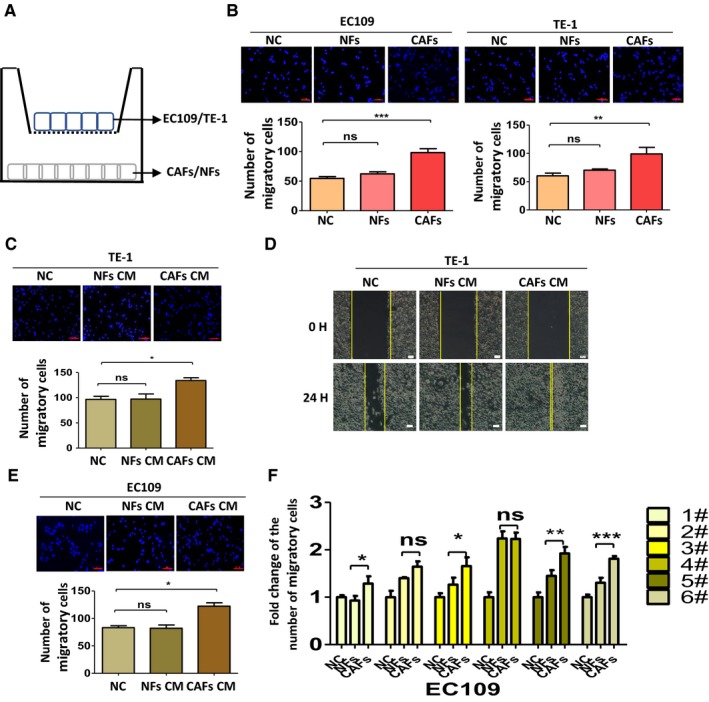
The conditioned medium (CM) of cancer‐associated fibroblasts (CAFs) enhanced the migration ability of esophageal squamous cell carcinoma cell lines. A, Schematic representation of the in‐direct coculture system. B, Cells cocultured with CAFs gained enhanced migration ability than that in control groups and normal fibroblasts (NFs) groups. C, The effect of CM of CAFs on the migration ability of TE‐1 by using Transwell migration assay. D, Cell migration ability was measured by a wound‐healing assay. The migration distances of the TE‐1 cells cultured with CAFs‐CM were increased. E, The effects of the CM of CAFs on the migration ability of EC109 cells in the Transwell assay. F, The effects of the CM of CAFs on the migration ability of EC109 were measured in the six paired stromal cells using Transwell migration assay. Results shown are representative of three independent experiments. Statistical significance *P < .05, **P < .01 and ***P < .001

In order to explore the impact of CAFs on the aggressive behavior of ESCC, we designed an in‐direct coculture system of fibroblasts and ESCC cell lines (Figure 2A). The ESCC cell lines (TE‐1 and EC109) were seeded in the upper chamber with 8‐μm‐pore size polycarbonate membranes in 24‐well Transwell plates, while NFs/CAFs were grown in the bottoms of the wells. After coculture for 48 hours, ESCC cell lines in CAFs groups showed enhanced migration abilities compared with the NFs groups and control groups (Figure 2B). This suggested that CAFs somehow promoted the aggressive ability of ESCC.

To further explore the effects of CAFs on the migration and invasion of ESCC, we cultured TE‐1 and EC109 with the CM of CAFs and NFs. The Transwell assay showed that CM of CAFs markedly increased the migration ability of TE‐1 cells, compared with the control group and NFs group (Figure 2C). Consistently, the migration distances of TE‐1 and EC109 cells cultured with CAFs‐CM were increased in wound‐healing assay (Figure 2D). Similar results were also observed in EC109 cells and were further corroborated by subsequent continuous tests in all six paired CAFs and NFs (Figure 2E,F). Those data revealed that CM of CAFs can substantially enhance the migration ability of TE‐1 and EC109, while CM of NFs only slightly promote those abilities of TE‐1 and EC109 compared with untreated group.

### 3.3 Isolation, characterization and internalization of exosomes

**Figure 3:**
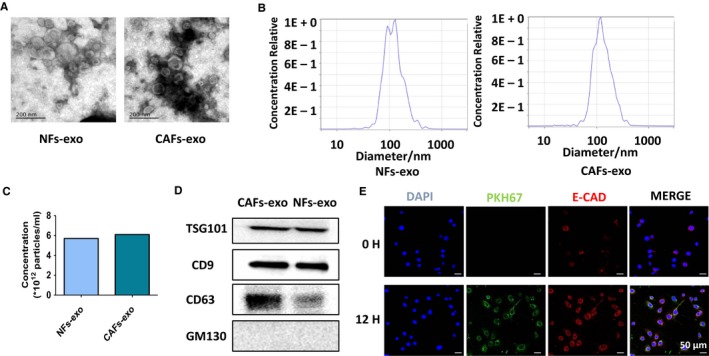
Isolation, characterization, and internalization of exosomes derived from primary stromal fibroblasts. A, Transmission electron microscopy images of cancer‐associated fibroblasts (CAFs)‐derived exosomes and normal fibroblasts (NFs)‐derived exosomes. B and C, The size and concentration of exosomes derived from CAFs and NFs examined by NTA. D, Western blot analyses of exosomal positive and negative markers (CD63, CD9, CD81, and GM130). E, Exosomes up‐taken experiment. The CAFs‐derived exosomes were labeled by PKH67 and incubated with TE‐1 cells and the green fluorescent protein‐tagged exosomes were taken up by the recipient cells after 12 h

Considering the above data alongside the increasing importance of exosomes in cellular dialogue, we speculated that CAFs‐derived exosomes may be involved in the above process. So, we isolated exosomes from NFs and CAFs by differential ultracentrifugation and the exosomes were firstly confirmed using TEM. The exosomes appear round, disc‐shaped structures with an average diameter of 50‐150 nm (Figure 3A). The concentration and size of the exosomes were further measured by NTA. It seemed that the CAFs may secrete more exosomes than NFs and both exosomes from CAFs and NFs present a main size of 80‐150 nm (Figure 3B,3). Besides, the significant expression of positive protein markers CD63, CD9, and TSG101 was observed in exosomes from NFs‐CM and CAFs‐CM while the negative protein marker GM130 was not detected (Figure 3D).

After identification, the exosomes were labeled by PKH67 and incubated with ESCC cell lines. As is expected, the green fluorescent protein‐tagged exosomes were found to be taken up by the recipient cells after 12 hours (Figure 3E). Taken together, these results clearly conformed that the exosomes we isolated are qualified and can be internalized by recipient cells.

### 3.4 CAFs‐derived exosomes promoted the aggressive behavior of ESCC cell lines

**Figure 4:**
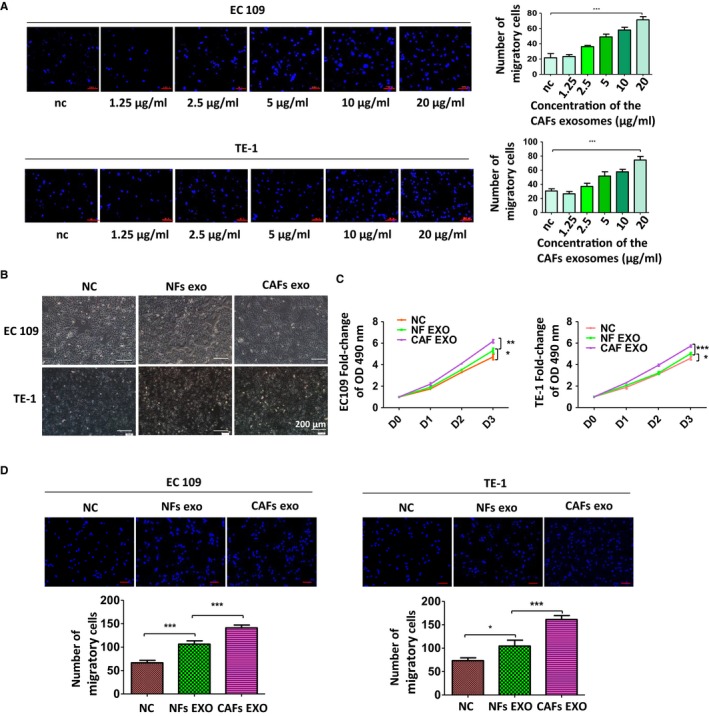
Cancer‐associated fibroblasts (CAFs)‐derived exosomes promoted the aggressive behavior of esophageal squamous cell carcinoma (ESCC) cell lines. A, CAFs‐derived exosomes could promote migration ability of ESCC cell lines with a dose‐dependent effect. B, CAFs‐derived exosomes did not change the morphological character of TE‐1 and EC109. C, Effects of CAFs‐derived exosomes on the proliferation of TE‐1 and EC109 cells was measured by MTS assay. D, Effects of CAFs‐derived exosomes on migration ability of ESCC cell lines. Results shown are representative of three independent experiments. Statistical significance *P < .05, **P < .01 and ***P < .001

In light of the crucial role of exosomes in the cell‐cell communication, we hypothesized that CAFs‐derived exosomes may contribute to the enhancement of aggressive behavior of ESCC. To test this hypothesis, we incubated TE‐1 and EC109 with gradually increased concentrations of exosomes derived from CAFs. The Transwell assay showed that CAFs‐derived exosomes could promote the migration ability of ESCC cell lines with a dose‐dependent effect (Figure 4A). In 20 μg/mL group, ESCC cell lines exhibits a markedly increased migration ability compared to the control groups. Therefore, the concentration of 20 μg/mL of exosomes was set for standard in the following experiments.

We then cultured TE‐1 and EC109 cells with exosomes from CAFs or NFs for 48h, and examined whether CAFs‐derived exosomes regulate the morphology and growth of ESCC cells. Apparent morphological changes of ESCC cells were not observed, even after treated with exosomes for 96 hours (Figure 4B). But CAFs‐derived exosomes significantly improved the proliferation ability of ESCC cells while the NFs‐derived exosomes enhanced the proliferation ability of ESCC cells moderately (Figure 4C). Also, ESCC cells cultured by CAFs‐derived exosomes gained higher migration ability than that in control group and in NFs group (Figure 4D). Collectively, our result illustrated that CAFs‐derived exosomes promotes some aggressive behavior of ESCC, at least in this experimental setting.

### 3.5 CAFs‐derived exosomes promoted proliferation and migration of ESCC via activation of SHH signaling pathway

**Figure 5:**
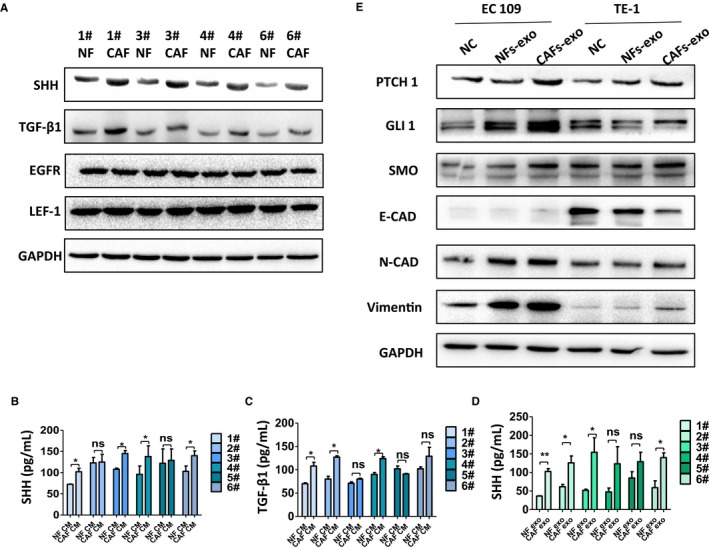
Cancer‐associated fibroblasts (CAFs)‐derived exosomes promoted proliferation and migration of esophageal squamous cell carcinoma via the activation of Sonic Hedgehog (SHH) signaling pathway. A, The expressions of SHH, TGFβ1, EGFR, and LEF‐1 in normal fibroblasts (NFs) and cancer‐associated fibroblasts (CAFs) were tested using WB. B and C, The expressions of SHH and TGF‐β1 in conditioned medium (CMs) of CAFs and NFs were tested using ELISA. The expression of TGF‐β1 and SHH in CMs of most CAFs was higher than in NFs group. D, The expression of SHH in exosomes from CAFs and NFs was measured by ELISA. E, The expressions of PTCH1, SMO, GLI1, N‐cadherin, vimentin, and E‐cadherin in TE‐1 and EC109 treated with or without exosomes were tested using WB

Despite the clear importance of CAFs‐derived exosomes to ESCC progression, the underlying mechanisms are extremely complex and not fully understood, with multiple factors at play. To identity the specific molecules in exosomes that contribute to the effects above, we examined the differential expression of several secretory proteins in the lysis solutions of NFs and CAFs. Previous studies^16^, ^31^, ^32^, ^33^, ^34^, ^35^, ^36^ indicated that TGFβ1, SHH, EGFR, and LEF‐1 may be transferred or modulated by exosomes and those proteins may play a part in the tumor development, so we tested the expression of those proteins in lysis solutions of NFs and CAFs. The expressions of those proteins in NFs and CAFs were tested using WB (Figure 5A), and CAFs expressed significantly more TGF‐β1 and SHH than NFs. There is no difference in the expression of EGFR and LEF‐1 between NFs and CAFs. We then compared the secretion of TGF‐β1 and SHH in CM of CAFs and NFs, ELISA showed that the expressions of TGF‐β1 and SHH in CMs of most CAFs were higher than in matched NFs group (Figure 5B,5).

It is noteworthy that the expression of SHH in exosomes of CAFs also elevated than in the exosomes of NFs (Figure 5D). Thus, it is tempting to speculate that exosomal SHH derived from CAFs may contribute to the aggressive behavior of ESCC. To test this hypothesis, we detected the expression of the downstream proteins in SHH signaling pathway in ESCC after treated with exosomes of NFs or CAFs. As shown in Figure 5E, CAFs‐derived exosomes increased the expressions of PTCH1, SMO, and GLI1 in ESCC cells, indicating that the SHH signaling pathway in ESCC cell lines was activated by the exogenous exosomes, compared with control group and NFs group. Noteworthy, the expressions of mesenchymal marker vimentin and N‐cadherin also increased and the expression of epithelial marker E‐cadherin decreased, which imply that the CAFs‐derived exosomes can facilitate the epithelial–mesenchymal transition (EMT) of ESCC cell lines. In summary, these results supported that CAFs‐derived exosomes may promote proliferation, migration of ESCC via the activation of SHH signaling pathway in vitro.

### 3.6 The phenotype changes of ESCC cells were neutralized by the inhibition of SHH signaling pathway

**Figure 6:**
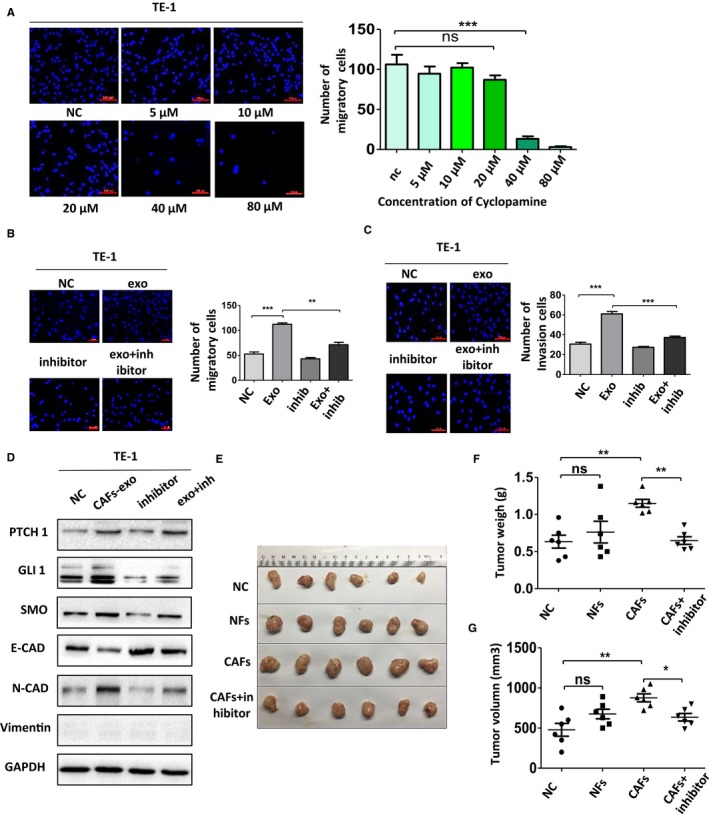
The phenotype changes were neutralized by the inhibition of Sonic Hedgehog signaling pathway in vitro and in vivo. A, The effects of Cyclopamine on the migration ability of TE‐1 cells. B and C, Cell migration and invasion ability was measured using Transwell assay. D, Western blot analysis of expressions of PTCH1, SMO, GLI1, N‐cadherin, vimentin, and E‐cadherin in TE‐1 cells after treated with Cyclopamine. E‐G, The appearance, weight, and volume of the subcutaneous tumors in mice

To further validate our findings, we investigated the relationship between exosomes‐induced phenotype changes and SHH pathway activation using a SMO inhibitor, Cyclopamine. TE‐1 cells were incubated with gradually increased concentrations of Cyclopamine, as shown in Figure 6A, and the number of migratory cells remains almost unchanged when the concentration of Cyclopamine is below 20 μmol/L and the number of migratory cells reduced sharply if the concentration is beyond 20 μmol/L. The concentration of 20 μmol/L was used in the following experiments.

Interestingly, Cyclopamine significantly neutralized the effects of CAFs‐derived exosomes. As predicted, Cyclopamine decreased the elevated number of migrating or invading cells in Transwell assay with or without Matrigel (Figure 6B,C). To further testify the role of SHH signaling in phenotype changes of ESCC cells, Cyclopamine, the inhibitor of SHH signaling pathway, was used to treat TE‐1 cells with or without the presence of CAFs‐derived exosomes. Then, the expressions of the relative proteins were examined. The elevated expressions of vimentin, N‐cadherin, PTCH1, SMO, GLI1, and downregulated expression of E‐cadherin in TE‐1 cells stimulated by CAFs‐derived exosomes were reversed (Figure 6D). Therefore, we proved that the inhibition of SHH pathway activation via blocking the activation of SMO could, at least in part, abolish the changes of proliferation, migration, and invasion induced by exosomes of CAFs.

We then used a subcutaneous xenograft tumor model in BALB/c nude mice to explore the effects of CAFs on the development of ESCC in vivo. Ec109 cells, with CAFs or NFs, were injected into nude mice, followed by the injection of Cyclopamine every other day. The results in vivo were consistent with those in vitro. As shown in Figure 6E‐G, tumor weight and volume in the CAFs group were higher than those in the NFs group and control group. Notably, the tendency could be reserved by Cyclopamine, concurring with expectation. In summary, these results indicated that CAFs facilitated ESCC growth in vivo and the block of SHH signaling could partly reverse this phenomenon.

## 4 DISCUSSION

The role of CAFs in the development of cancer has attracted wide attention in recent years. Here, we presented evidence that CAFs play a key role in the proliferation and migration of ESCC. CAFs‐secreted exosomes can be internalized by tumor cells and enhance the aggressive behavior of ESCC. Moreover, our results indicated that CAFs‐secreted exosomes, rich in SHH, facilitate proliferation, and migration of ESCC via the activation of SHH signaling pathway.

To date, the isolation and cultures of stromal fibroblasts from primary tissues are the single most important approach to study the features of CAFs and their influence on tumor cells. Immortalized fibroblasts cell lines or primary stromal fibroblasts passed more than 10 times may possess different properties, and consequently compromise subsequent results. In this study, we successfully isolated six paired NFs and CAFs from ESCC tissues and adjacent esophageal tissues. Those fibroblasts isolated from ESCC tissues were verified to present the features of CAFs, with a higher expression of a‐SMA than in NFs, and both kinds of stromal fibroblasts used in our experiments were no more than eight passages. Accumulated evidence indicates that CAFs could promote aggressive behavior of variety of cancer cells. Donnarumma et al found that breast cancer cells cultured by the CAFs‐derived exosomes exhibited a significantly increased ability to form mammospheres, anchorage‐independent cell growth and higher expression of stem cell and EMT markers.^37^ It is also reported that, in breast cancer, the activated stromal fibroblasts could contribute to the VEGF‐independent angiogenesis via the miR‐205/YAP1 signaling.^38^ In HCC, Liu et al showed that CAFs promote HCC metastasis through the activation of Hedgehog and TGF‐β pathways via chemokines.^9^ Collectively, these results revealed that CAFs‐tumor cell crosstalk facilitates the progression of tumors. Unfortunately, data on the importance of CAFs in ESCC are sparse at best, and the specific molecules and mechanisms that modulate ESCC development remain largely unknown. Here, in agreement with those data, our coculture system demonstrated that CAFs can somehow affect the phenotypes of esophageal cancer cells, and ESCC cells treated with CM of CAFs acquired better migration and invasion ability, which indicated that biological molecules secreted by CAFs can enhance the aggressive behaviors of ESCC cancer cells.

Recent studies showed that exosomes, targeting genetic information such as mRNAs and proteins, act as a mediator of genetic information transmission between different cells. Molecules such as proteins, mRNAs can be carried by those extracellular vesicles to other cells, and thereby lead to the changes of gene expression and phenotype of recipient cells. Deng et al revealed that Exosomal LINC00461 could regulate the expression of microRNA/BCL‐2 to enhance the proliferation ability and inhibit apoptosis in multiple myeloma.^39^ Exosome‐transmitted circular RNAs of gastric tumor can modulate the miR‐133/PRDM16 pathway to promote white adipose browning.^40^ Exosomes from platelet‐rich plasma can substantially improve the proliferation and migration ability of endothelial and mesenchymal cells and induce the angiogenesis and reepithelialization of chronic wounds.^41^ All those studies together illustrated that exosomal bioactive molecules including mRNA, microRNAs, and proteins can modulate the activity of recipient cells. In this study, we first isolated exosomes from NFs and CAFs and verified that the CAFs‐derived exosomes can be incorporated into the tumor cells and affect the phenotypes of ESCC cancer cells. In particular, we found that cells cultured with CAFs‐secreted exosomes gained increased proliferation, migration, and invasion ability, with a dose‐dependent effect. These results indicated that CAFs‐exosomes play a potent cell proliferation and migration‐promoting role during progression of ESCC, and it will be of great value to identify the specific determinants and the subsequent signaling pathways.

Several signaling pathways can regulate cancer cell proliferation, migration, and invasion, however, it is unclear which one is directly involved in this phenomenon. In light of significance of TGFβ1, SHH, EGFR, and LEF‐1 in the proliferation and migration of cancer cells,^22^, ^42^, ^43^, ^44^, ^45^ we examined the differential expression of those proteins in the cell lysis solution of NFs and CAFs and paid our attention to the different expression of SHH. As previously reported, abnormal activation of Hedgehog signaling pathway was positively correlated with the clinical manifestations, lymph node metastasis, and overall survival rate of many cancers.^46^, ^47^ Strikingly, SHH signaling pathway is not merely a cell‐intrinsic pathway that regulates the activity of the cancer cell, but is also modulated by the extrinsic microenvironment.^48^, ^49^ Recent studies showed that exosomes can act as natural vehicles for delivering protein of SHH. Qi et al found that adipocyte‐derived exosomes containing SHH lead to the M1 polarization of macrophages, which promotes insulin resistance via the Ptch/PI3K pathway in adipocytes.^32^ Neha revealed that vertebrate Hedgehog is secreted on two types of extracellular vesicles with different functions.^31^ Herein, we identified that the expression of SHH elevated in cell lysis solution, CM and exosomes of CAFs compared to the NFs. And the CAFs‐derived exosomes could efficiently enhance the proliferation and migration of ESCC in vitro. Besides, CAFs can facilitate ESCC growth in vivo. Interestingly, the expressions of PTCH1, SMO, and GLI1, downstream proteins of SHH pathway, increased and the inhibition of SHH pathway via blocking the activation of SMO could, at least in part, reversed this phenomenon. Those results were observed both in vitro and in vivo. Together, these results indicated that CAFs‐derived exosomes promote proliferation and migration of ESCC via the activation of SHH signaling pathway.

Recent studies revealed that the activation of SHH signaling not only contributes to the progression of ESCC, but influences the therapeutic outcomes and prognosis of patients with esophageal cancer. Some scholars demonstrated that the expression of SHH are associated with the survival outcome of the neoadjuvant chemoradiotherapy (nCRT) in patients with ESCC,^50^ and the expression of PTCH1 and Gli1 may be significantly associated with ESCC resistance to chemoradiotherapy.^51^ Furthermore, inhibition of Hedgehog signaling can regulate radiation sensitivity in mouse xenograft models of human esophageal cancer.^52^ So, targeting the SHH pathway may become a novel approach to prevent or inhibit the progression of ESCC, this is consistent with our point of view. In our study, we found that inhibiting the exosomal transfer of SHH from CAFs to cancer cells can influence the progression of ESCC in vivo and in vitro. Besides, pioneering studies have implied that intervention of the Hedgehog pathway may prevent the progression of Barrett's esophagus to invasive esophageal adenocarcinoma,^53^ and more researches are needed to clarify its effectiveness.

In most studies, the binding between SHH and PTCH1 results in the derepression of SMO, consequently activating the SHH signaling and leading to the translocation of the GLI1 to the nucleus.^54^, ^55^ Nuclear GLI1 can regulate the expressions of many genes including PTCH1 and Gli1. In our study, we observed an increasing expression of GLI1 as well as the PTCH1 and SMO, which is not identical to the classic SHH signaling. Previous studies have indicated that in addition to the classical signaling axis, the SHH signaling can also be triggered by nonclassical pathways. Nonclassical SHH signaling mainly refers to the activation of this pathway from PTCH1/SMO but not involving the expression of GLI1, or the overexpression of GLI1 not triggered by SHH.^56^, ^57^ So, the increasing expression of PTCH1, SMO, and GLI1 may also be triggered by other factors in CAFs‐derived exosomes aside from SHH, which need to be further investigated.

It should be noted that the exosomal SHH is not the single molecular determinants that promote the aggressive behavior of the ESCC cells. In our study, the expression of TGF‐β1 in CM of CAFs also elevated compared to that in NFs, and the function of TGF‐β1 in tumor progression has been well recognized. We believe that CAFs‐exosomes could facilitate the aggressive behavior of the ESCC cells via various signaling pathways among which the SHH signaling may be of great importance.

## 5 CONCLUSION

In summary, our findings demonstrated that CAFs‐secreted exosomes could promote the growth and metastatic potential of ESCC through the activation of SHH signaling pathway. In addition to its biological importance, our study also suggested that preventing or inhibiting the exosomal transfer of SHH from CAFs to cancer cells may be a new strategy for the treatment of ESCC.

## COMPETING INTERESTS

The authors have declared that no competing interest exists.

## References

[1] Chen W , Zheng R , Baade PD , et al. Cancer statistics in China, 2015. CA Cancer J Clin. 2016;66(2):115‐132. 26808342 10.3322/caac.21338

[2] Abbas G , Krasna M . Overview of esophageal cancer. Ann Cardiothorac Surg. 2017;6(2):131‐136. 28447001 10.21037/acs.2017.03.03 PMC5387155

[3] Liu J , Xie X , Zhou C , Peng S , Rao D , Fu J . Which factors are associated with actual 5‐year survival of oesophageal squamous cell carcinoma? Eur J Cardiothorac Surg. 2012;41(3):e7‐e11. 22219482 10.1093/ejcts/ezr240

[4] Hui L , Chen Y . Tumor microenvironment: sanctuary of the devil. Cancer Lett. 2015;368(1):7‐13. 26276713 10.1016/j.canlet.2015.07.039

[5] Soysal SD , Tzankov A , Muenst SE . Role of the tumor microenvironment in breast cancer. Pathobiology. 2015;82(3‐4):142‐152. 26330355 10.1159/000430499

[6] Bussard KM , Mutkus L , Stumpf K , Gomez‐Manzano C , Marini FC . Tumor‐associated stromal cells as key contributors to the tumor microenvironment. Breast Cancer Res. 2016;18(1):84. 27515302 10.1186/s13058-016-0740-2 PMC4982339

[7] Chen J , Yang P , Xiao Y , et al. Overexpression of alpha‐sma‐positive fibroblasts (CAFs) in nasopharyngeal carcinoma predicts poor prognosis. J Cancer. 2017;8(18):3897‐3902. 29151978 10.7150/jca.20324 PMC5688944

[8] Yu Y , Xiao CH , Tan LD , Wang QS , Li XQ , Feng YM . Cancer‐associated fibroblasts induce epithelial‐mesenchymal transition of breast cancer cells through paracrine TGF‐beta signalling. Br J Cancer. 2014;110(3):724‐732. 24335925 10.1038/bjc.2013.768 PMC3915130

[9] Liu J , Chen S , Wang W , et al. Cancer‐associated fibroblasts promote hepatocellular carcinoma metastasis through chemokine‐activated hedgehog and TGF‐beta pathways. Cancer Lett. 2016;379(1):49‐59. 27216982 10.1016/j.canlet.2016.05.022

[10] Trams EG , Lauter CJ , Norman Salem JR , Heine U . Exfoliation of membrane ecto‐enzymes in the form of micro‐vesicles. Biochim Biophys Acta. 1981;645(1):63‐70. 6266476 10.1016/0005-2736(81)90512-5

[11] Kharaziha P , Ceder S , Li Q , Panaretakis T . Tumor cell‐derived exosomes: a message in a bottle. Biochim Biophys Acta. 2012;1826(1):103‐111. 22503823 10.1016/j.bbcan.2012.03.006

[12] Oosthuyzen W , Sime NEL , Ivy JR , et al. Quantification of human urinary exosomes by nanoparticle tracking analysis. J Physiol. 2013;591(23):5833‐5842. 24060994 10.1113/jphysiol.2013.264069 PMC3872755

[13] Street JM , Barran PE , Mackay CL , et al. Identification and proteomic profiling of exosomes in human cerebrospinal fluid. J Transl Med. 2012;10:5. 22221959 10.1186/1479-5876-10-5 PMC3275480

[14] Lässer C , Alikhani VS , Ekström K , et al. Human saliva, plasma and breast milk exosomes contain RNA: uptake by macrophages. J Transl Med. 2011;9:9. 21235781 10.1186/1479-5876-9-9 PMC3033821

[15] Li W , Zhang X , Wang J , et al. TGFbeta1 in fibroblasts‐derived exosomes promotes epithelial‐mesenchymal transition of ovarian cancer cells. Oncotarget. 2017;8(56):96035‐96047. 29221185 10.18632/oncotarget.21635 PMC5707079

[16] Zhang H , Deng T , Liu R , et al. Exosome‐delivered EGFR regulates liver microenvironment to promote gastric cancer liver metastasis. Nat Commun. 2017;8:15016. 28393839 10.1038/ncomms15016 PMC5394240

[17] Li X , Yang Z . Regulatory effect of exosome on cell apoptosis. Zhong Nan Da Xue Xue Bao Yi Xue Ban. 2017;42(2):215‐220. 28255126 10.11817/j.issn.1672-7347.2017.02.016

[18] Cao Y , Lin S‐H , Wang Y , Chin YE , Kang L , Mi J . Glutamic pyruvate transaminase GPT2 promotes tumorigenesis of breast cancer cells by activating Sonic Hedgehog signaling. Theranostics. 2017;7(12):3021‐3033. 28839461 10.7150/thno.18992 PMC5566103

[19] Taylor MD , Liu L , Raffel C , et al. Mutations in SUFU predispose to medulloblastoma. Nat Genet. 2002;31(3):306‐310. 12068298 10.1038/ng916

[20] Berman DM , Karhadkar SS , Maitra A , et al. Widespread requirement for Hedgehog ligand stimulation in growth of digestive tract tumours. Nature. 2003;425(6960):846‐851. 14520411 10.1038/nature01972

[21] Huaitong X , Yuanyong F , Yueqin T , Peng Z , Wei S , Kai S . Microvesicles releasing by oral cancer cells enhance endothelial cell angiogenesis via Shh/RhoA signaling pathway. Cancer Biol Ther. 2017;18(10):783‐791. 28886265 10.1080/15384047.2017.1373213 PMC5678693

[22] Zhang F , Ren CC , Liu L , et al. SHH gene silencing suppresses epithelial‐mesenchymal transition, proliferation, invasion, and migration of cervical cancer cells by repressing the Hedgehog signaling pathway. J Cell Biochem. 2018;119(5):3829‐3842. 28941302 10.1002/jcb.26414

[23] Jeng KS , Jeng CJ , Jeng WJ , et al. Sonic Hedgehog signaling pathway as a potential target to inhibit the progression of hepatocellular carcinoma. Oncol Lett. 2019;18(5):4377‐4384. 31611946 10.3892/ol.2019.10826 PMC6781692

[24] Giroux‐Leprieur E , Costantini A , Ding V , He B . Hedgehog signaling in lung cancer: from oncogenesis to cancer treatment resistance. Int J Mol Sci. 2018;19(9):2835. 10.3390/ijms19092835 PMC6165231 30235830

[25] Ma X , Sheng T , Zhang Y , et al. Hedgehog signaling is activated in subsets of esophageal cancers. Int J Cancer. 2006;118(1):139‐148. 16003737 10.1002/ijc.21295

[26] Yang L , Wang LS , Chen XL , et al. Hedgehog signaling activation in the development of squamous cell carcinoma and adenocarcinoma of esophagus. Int J Biochem Mol Biol. 2012;3(1):46‐57. 22509480 PMC3325770

[27] Wang L , Jin JQ , Zhou Y , Tian Z , Jablons DM , He B . Gli is activated and promotes epithelial‐mesenchymal transition in human esophageal adenocarcinoma. Oncotarget. 2018;9(1):853‐865. 29416661 10.18632/oncotarget.22856 PMC5787518

[28] Mori Y , Okumura T , Tsunoda S , Sakai Y , Shimada Y . Gli‐1 expression is associated with lymph node metastasis and tumor progression in esophageal squamous cell carcinoma. Oncology. 2006;70(5):378‐389. 17179732 10.1159/000098111

[29] Saito T , Mitomi H , Imamhasan A , et al. PTCH1 mutation is a frequent event in oesophageal basaloid squamous cell carcinoma. Mutagenesis. 2015;30(2):297‐301. 25395299 10.1093/mutage/geu072

[30] Edge SB , Compton CC . The American Joint Committee on Cancer: the 7th edition of the AJCC cancer staging manual and the future of TNM. Ann Surg Oncol. 2010;17(6):1471‐1474. 20180029 10.1245/s10434-010-0985-4

[31] Vyas N , Walvekar A , Tate D , et al. Vertebrate Hedgehog is secreted on two types of extracellular vesicles with different signaling properties. Sci Rep. 2014;4:7357. 25483805 10.1038/srep07357 PMC4258658

[32] Song M , Han LU , Chen F‐F , et al. Adipocyte‐derived exosomes carrying Sonic Hedgehog mediate M1 macrophage polarization‐induced insulin resistance via Ptch and PI3K pathways. Cell Physiol Biochem. 2018;48(4):1416‐1432. 30064125 10.1159/000492252

[33] Borges FT , Melo SA , Özdemir BC , et al. TGF‐beta1‐containing exosomes from injured epithelial cells activate fibroblasts to initiate tissue regenerative responses and fibrosis. J Am Soc Nephrol. 2013;24(3):385‐392. 23274427 10.1681/ASN.2012101031 PMC3582210

[34] Zhao X , Wu X , Qian M , Song Y , Wu D , Zhang W . Knockdown of TGF‐beta1 expression in human umbilical cord mesenchymal stem cells reverts their exosome‐mediated EMT promoting effect on lung cancer cells. Cancer Lett. 2018;428:34‐44. 29702191 10.1016/j.canlet.2018.04.026

[35] Raulf N , Lucarelli P , Thavaraj S , et al. Annexin A1 regulates EGFR activity and alters EGFR‐containing tumour‐derived exosomes in head and neck cancers. Eur J Cancer. 2018;102:52‐68. 30142511 10.1016/j.ejca.2018.07.123

[36] Seo M , Kim JC , Kim HK , et al. A novel secretory vesicle from deer antlerogenic mesenchymal stem cell‐conditioned media (DaMSC‐CM) promotes tissue regeneration. Stem Cells Int. 2018;2018:3891404. 29765409 10.1155/2018/3891404 PMC5889873

[37] Donnarumma E , Fiore D , Nappa M , et al. Cancer‐associated fibroblasts release exosomal microRNAs that dictate an aggressive phenotype in breast cancer. Oncotarget. 2017;8(12):19592‐19608. 28121625 10.18632/oncotarget.14752 PMC5386708

[38] Du Y‐E , Tu G , Yang G , et al. MiR‐205/YAP1 in activated fibroblasts of breast tumor promotes VEGF‐independent angiogenesis through STAT3 signaling. Theranostics. 2017;7(16):3972‐3988. 29109792 10.7150/thno.18990 PMC5667419

[39] Deng M , Yuan H , Liu S , Hu Z , Xiao H . Exosome‐transmitted LINC00461 promotes multiple myeloma cell proliferation and suppresses apoptosis by modulating microRNA/BCL‐2 expression. Cytotherapy. 2019;21(1):96‐106. 30409700 10.1016/j.jcyt.2018.10.006

[40] Zhang H , Zhu L , Bai M , et al. Exosomal circRNA derived from gastric tumor promotes white adipose browning by targeting the miR‐133/PRDM16 pathway. Int J Cancer. 2019;144(10):2501‐2515. 30412280 10.1002/ijc.31977

[41] Guo S‐C , Tao S‐C , Yin W‐J , Qi X , Yuan T , Zhang C‐Q . Exosomes derived from platelet‐rich plasma promote the re‐epithelization of chronic cutaneous wounds via activation of YAP in a diabetic rat model. Theranostics. 2017;7(1):81‐96. 28042318 10.7150/thno.16803 PMC5196887

[42] Wu DM , Deng SH , Liu T , Han R , Zhang T , Xu Y . TGF‐beta‐mediated exosomal lnc‐MMP2‐2 regulates migration and invasion of lung cancer cells to the vasculature by promoting MMP2 expression. Cancer Med. 2018;7(10):5118‐5129. 30256540 10.1002/cam4.1758 PMC6198203

[43] Tian X , Fei Q , Du M , et al. miR‐130a‐3p regulated TGF‐beta1‐induced epithelial‐mesenchymal transition depends on SMAD4 in EC‐1 cells. Cancer Med. 2019;8(3):1197‐1208. 30741461 10.1002/cam4.1981 PMC6434193

[44] Watanabe H , Okauchi S , Miyazaki K , Satoh H , Hizawa N . Factors associated with distant metastasis in EGFR‐mutated non‐small cell lung cancer patients: logistic analysis. In Vivo. 2019;33(4):1369‐1372. 31280232 10.21873/invivo.11613 PMC6689373

[45] Kobayashi W , Ozawa M . The epithelial‐mesenchymal transition induced by transcription factor LEF‐1 is independent of beta‐catenin. Biochem Biophys Rep. 2018;15:13‐18. 29998192 10.1016/j.bbrep.2018.06.003 PMC6038150

[46] Yoshizaki A , Nakayama T , Naito S , Wen CY , Sekine I . Expressions of sonic hedgehog, patched, smoothened and Gli‐1 in human intestinal stromal tumors and their correlation with prognosis. World J Gastroenterol. 2006;12(35):5687‐5691. 17007023 10.3748/wjg.v12.i35.5687 PMC4088171

[47] Pietanza MC , Litvak AM , Varghese AM , et al. A phase I trial of the Hedgehog inhibitor, sonidegib (LDE225), in combination with etoposide and cisplatin for the initial treatment of extensive stage small cell lung cancer. Lung Cancer. 2016;99:23‐30. 27565909 10.1016/j.lungcan.2016.04.014 PMC5427482

[48] Handrigan GR , Richman JM . Autocrine and paracrine Shh signaling are necessary for tooth morphogenesis, but not tooth replacement in snakes and lizards (Squamata). Dev Biol. 2010;337(1):171‐186. 19850027 10.1016/j.ydbio.2009.10.020

[49] Shaw A , Gipp J , Bushman W . Exploration of Shh and BMP paracrine signaling in a prostate cancer xenograft. Differentiation. 2010;79(1):41‐47. 19773112 10.1016/j.diff.2009.08.009 PMC2787832

[50] Honing J , Pavlov KV , Mul VEM , et al. CD44, SHH and SOX2 as novel biomarkers in esophageal cancer patients treated with neoadjuvant chemoradiotherapy. Radiother Oncol. 2015;117(1):152‐158. 26364884 10.1016/j.radonc.2015.08.031

[51] Zhu W , You Z , Li T , et al. Correlation of hedgehog signal activation with chemoradiotherapy sensitivity and survival in esophageal squamous cell carcinomas. Jpn J Clin Oncol. 2011;41(3):386‐393. 21127038 10.1093/jjco/hyq217

[52] Teichman J , Dodbiba L , Thai H , et al. Hedgehog inhibition mediates radiation sensitivity in mouse xenograft models of human esophageal adenocarcinoma. PLoS ONE. 2018;13(5):e0194809. 29715275 10.1371/journal.pone.0194809 PMC5929523

[53] Kelly RJ , Ansari AM , Miyashita T , et al. Targeting the Hedgehog pathway using itraconazole to prevent progression of Barrett's esophagus to invasive esophageal adenocarcinoma. Ann Surg. 2019. 10.1097/SLA.0000000000003455 PMC8147663 31290765

[54] Walter K , Omura N , Hong S‐M , et al. Overexpression of smoothened activates the sonic hedgehog signaling pathway in pancreatic cancer‐associated fibroblasts. Clin Cancer Res. 2010;16(6):1781‐1789. 20215540 10.1158/1078-0432.CCR-09-1913 PMC2846609

[55] Li S , Li S , Wang B , Jiang J . Hedgehog reciprocally controls trafficking of Smo and Ptc through the Smurf family of E3 ubiquitin ligases. Sci Signal. 2018;11(516):eaan8660. 29438012 10.1126/scisignal.aan8660 PMC6404747

[56] Rimkus T , Carpenter R , Qasem S , Chan M , Lo H‐W . Targeting the Sonic Hedgehog signaling pathway: review of smoothened and GLI inhibitors. Cancers. 2016;8(2):22. 10.3390/cancers8020022 PMC4773745 26891329

[57] Ke Z , Caiping S , Qing Z , Xiaojing W . Sonic hedgehog‐Gli1 signals promote epithelial‐mesenchymal transition in ovarian cancer by mediating PI3K/AKT pathway. Med Oncol. 2015;32(1):368. 25432698 10.1007/s12032-014-0368-y

